# Visual short-term memory load modulates the early attention and perception of task-irrelevant emotional faces

**DOI:** 10.3389/fnhum.2015.00490

**Published:** 2015-09-03

**Authors:** Ping Yang, Min Wang, Zhenlan Jin, Ling Li

**Affiliations:** Key Laboratory for NeuroInformation of Ministry of Education, School of Life Science and Technology, University of Electronic Science and Technology of ChinaChengdu, China

**Keywords:** task-irrelevant emotional distraction, VSTM maintenance, P1, N1, P300, PSW, NSW

## Abstract

The ability to focus on task-relevant information, while suppressing distraction, is critical for human cognition and behavior. Using a delayed-match-to-sample (DMS) task, we investigated the effects of emotional face distractors (positive, negative, and neutral faces) on early and late phases of visual short-term memory (VSTM) maintenance intervals, using low and high VSTM loads. Behavioral results showed decreased accuracy and delayed reaction times (RTs) for high vs. low VSTM load. Event-related potentials (ERPs) showed enhanced frontal N1 and occipital P1 amplitudes for negative faces vs. neutral or positive faces, implying rapid attentional alerting effects and early perceptual processing of negative distractors. However, high VSTM load appeared to inhibit face processing in general, showing decreased N1 amplitudes and delayed P1 latencies. An inverse correlation between the N1 activation difference (high-load minus low-load) and RT costs (high-load minus low-load) was found at left frontal areas when viewing negative distractors, suggesting that the greater the inhibition the lower the RT cost for negative faces. Emotional interference effect was not found in the late VSTM-related parietal P300, frontal positive slow wave (PSW) and occipital negative slow wave (NSW) components. In general, our findings suggest that the VSTM load modulates the early attention and perception of emotional distractors.

## Introduction

Visual short-term memory (VSTM) was proposed as a broadly-defined limited-capacity cognitive system for temporarily maintaining representations of external information to support human cognition and behavior (Baddeley, 2003). The central executive, which is the most important component of VSTM, is responsible for allocating attention to task-relevant information while suppressing task-irrelevant information (Norman and Shallice, 1986; Baddeley, 2003). This function is critical for us in the real world, (e.g., driving) where we need to focus our attention and devote our processing resources to a goal-related task, while ignoring other distracting information. However, numerous studies have demonstrated that task-irrelevant information impairs the performance of VSTM and reduces its capacity (Dolcos and McCarthy, 2006; Erk et al., 2007; Anticevic et al., 2010; MacNamara et al., 2012). The active maintenance of task-relevant information and the inhibition of task-irrelevant information have been linked to the dorsolateral prefrontal cortex (DLPFC) and lateral parietal cortex (LPC; Chafee and Goldman-Rakic, 2000; Sakai et al., 2002; Jha et al., 2004; Dolcos and McCarthy, 2006). In addition, Iordan et al. (2013) demonstrated that the ventrolateral prefrontal cortex (VLPFC) is also linked to coping with emotional distraction.

According to the valence of emotional stimuli, emotional stimuli were categorized as positive, negative and neutral. Numbers of studies have reported valence-related performance or activity of emotional stimuli (Smith et al., 2003; Erk et al., 2007; Luo et al., 2010; Kong et al., 2013). Emotional stimuli capture attention easily and induce a reallocation of resources, which enables a rapid evaluation and decision making in the service of survival (Dolcos and McCarthy, 2006; Petroni et al., 2011). For example, negative emotional stimuli are processed rapidly and automatically by the amygdala and ventral striatum structures, and this may be valuable for detecting and recognizing potential threats and dangers (Pessoa, 2008; Stout et al., 2013). However, if the emotional stimuli serve as distractors, they may capture the limited attention or processing resources, and thus impair cognitive performance of functions such as VSTM. Several studies have found that emotional stimuli serving as distractors disturb VSTM function in all of three phases of VSTM, including encoding, maintaining, and retrieving relevant information (Dolcos et al., 2008; Clapp et al., 2010; Ziaei et al., 2014). However, encoding-distraction and maintaining-distraction tasks are different. For example, the encoding-distraction task requires selective attention and perception of the task-relevant stimuli, when ignoring the simultaneous presentation of distractors (Ziaei et al., 2014). Whereas, the maintaining-distraction task exhibits a sudden onset of distractors after the encoding of task-relevant stimuli, and the distractors may reopen the perception gate and capture attention (McNab and Dolan, 2014). Using emotional faces as encoding-distractors, Stout et al. found that negative face distractors gained unnecessary access to VSTM and were inefficiently filtered, when compared with neutral face distractors (Stout et al., 2013). On the other hand when emotional faces are used as maintaining-distractors, negative distractors show increased activity in ventral regions and decreased activity in dorsal regions, resulting in impaired VSTM performance when compared with neutral distractors (Dolcos and McCarthy, 2006). Furthermore, McNab and Dolan (2014) compared the VSTMC of three conditions: no distraction, encoding distraction and delay distraction, and found a dissociative result across the three conditions. This study used hierarchical regression analysis, where the predicted VSTMC was the no distractor condition, and the regressors were the VSTMC of encoding and delay distraction conditions. The authors found that both *k* values at the encoding and delay distraction conditions were uniquely positively associated with predicted VSTMC, suggesting a separate mechanism of distractor filtering in the encoding and delay phase of VSTM (McNab and Dolan, 2014). In summary, negative distractors gained preferential processing and impaired task-related VSTM compared with neutral distractors, whether in the encoding or in the maintaining distraction task. In the present study, we examine whether the emotional maintaining-distractors interfere with the task-relevant information maintained in VSTM, and whether the interference effect is valence-dependent.

Studies show that due to the limited capacity of VSTM, the emotional interference on VSTM task is reduced with the increasing cognitive load. Behavioral studies have found that maintaining-distractors slowed down the VSTM reaction times (RTs) and increased the error rates (Kim et al., 2005), and this effect was significantly bigger when viewing negative or positive compared with neutral distractors (Miendlarzewska et al., 2013). However, this interference was diminished after increases in task difficulty, suggesting that negative distractors may have the equal interference effect as neutral distractors during VSTM maintenance in a high VSTM load task (Anticevic et al., 2010; Miendlarzewska et al., 2013). Moreover, the activity, induced by emotional distractors, in the amygdala and ventral striatum was reduced in the high-load task, implying insufficient VSTM capacity for emotional processing (Zald, 2003; Erk et al., 2007). An alternative interpretation could be that high cognitive load demands enhance top-down control, which in turn acts to inhibit emotional processing, resulting in decreased emotion-related interference (Clarke and Johnstone, 2013).

According to Lavie’s load theory of attention, there is an “early and late” selective attention mechanism that may account for the interaction between emotional distraction and perceptual load. The early selection view suggests that as the perceptual load increases, there is no sufficient capacity to perceive task-irrelevant distractors, resulting in better task-related performance and less distractibility, while the task-irrelevant distractors are perceived when the perceptual load is low. In recent years, Konstantinou and Lavie (2013) have expanded the theory to VSTM and proposed that VSTM load should have the same effect as perceptual load in reducing visual-representation capacity to perceive distractors. The late selection view suggests that even when the perceptual load is low and task-irrelevant distractors are perceived, a more active cognitive control mechanism, with functional links to WM (and perhaps also VSTM), can modulate top-down control responsible for maintaining task-relevant information and suppressing the distractors in later stages. However if cognitive control is loaded by a previous task (e.g., memory maintenance), performance on a subsequent task (e.g., visual search) will suffer from a worsened ability to inhibit task-irrelevant distractors (Lavie et al., 2004; Konstantinou and Lavie, 2013). In summary, early and late selection are associated with high and low perceptual load conditions, respectively. Lavie’s load theory of attention solves the early and late selection debate and demonstrates that early and late selection are two processing levels in a hybrid model of attention, instead of two separate mechanisms.

To investigate early and late selection, evoked-related potential (ERP) measurements with high time-resolution can be used to track the time course of interaction between emotional distractors and VSTM load. To this end we used the distractor perception-related P1/N1 (early ERP component), the VSTM-related P300 and the sustained slow wave (late ERP component). We examined how the VSTM load modulates the emotional distraction during early and late selection. We also examined the valence-related interference effect from distractors on task-relevant information maintained in VSTM.

The ERP P1 component, which peaks around 80–150 ms over occipital areas is associated with attention allocation (Clark and Hillyard, 1996). For example, P1 is larger for stimuli that are located in an attended location compared to stimuli that are located in an unattended location (Hillyard et al., 1998). Combining the dipole localization and MRI method, Di Russo et al. (2002) argued that P1 component is generated in the extrastriate cortex. Specifically, previous ERP studies have demonstrated that the P1 component is linked to early perceptual processing of emotional faces (Smith et al., 2003), showing enhanced amplitudes in the presence of negative faces (such as fear and angry faces) vs. neutral or positive faces (Batty and Taylor, 2003; Palermo and Rhodes, 2007; Olofsson et al., 2008; Rellecke et al., 2012). It has been suggested that negative stimuli are processed rapidly and automatically via a fast magnocellular route (Vuilleumier, 2005). Recently, Valdés-Conroy et al. (2014) found that angry faces elicited larger P1 amplitude compared with neutral faces, whether the face was task-relevant or task-irrelevant. In addition, Clapp and Gazzaley have suggested that the early P1 component (marker of selective attention for faces) can be influenced by top-down modulation of visual processing, showing longer latencies and smaller P1 amplitudes for ignored compared to attended faces (Clapp et al., 2010). However, as the VSTM load increases (memorization of four items), the enhancement indices of face significantly decreases, suggesting that increasing VSTM load exhausts the limited top-down attentional resources, thus resulting in diminished early activity modulation (Gazzaley et al., 2005, 2008; Gazzaley, 2011). In addition, numerous ERP studies have found that N1 component (80–150 ms post face onset) over frontal areas was related to facial expression processing, showing that N1 amplitude for negative faces was larger compared with neutral (Eimer and Holmes, 2002; Holmes et al., 2003; Wessing et al., 2013), and happy faces (Luo et al., 2010). This suggests that the orbitofrontal cortex serves as a rapid detector of emotional stimuli (Rippon et al., 2001; Luo et al., 2010). Santos et al. (2008) further suggested that orbitofrontal cortex could modulate the extrastriate cortex activity through a top-down attentional alerting mechanism to generate rapid responses to potentially threatening stimuli. Thus the P1 and N1 may be relevant measures for the study of early attention and perception of task-irrelevant emotional distractors.

Regarding late ERP components, we used the VSTM-related P300, which is a positive wave observed at central-parietal electrode sites approximately 300–650 ms post-stimulus onset. P300 is thought to reflect the update of VSTM and its amplitude is associated with cognitive task demands (Donchin and Coles, 1988). Numerous studies have found that the P300 amplitude decreases when more items are maintained in VSTM (Kok, 2001; Watter et al., 2001; Busch and Herrmann, 2003). The P300 amplitude is thought to reflect the demands of “perceptual-central” resources. Thus, as more items are maintained in VSTM, less resources remain and the P300 amplitude decreases (Kramer and Spinks, 1991), suggesting that the P300 may be an index of VSTM demands. We examined whether the VSTM-related P300 component is influenced by emotional distraction. Specifically we tested the hypothesis that emotional distractors, containing negative and positive faces, are inefficiently filtered in the early stages of perception, gaining unnecessary access to VSTM. We thus, expected the P300 amplitude to decrease for emotional faces compared with neutral faces.

In addition, we measured the sustained slow wave, which is a long-duration ERP component during the VSTM maintenance period (Ruchkin et al., 1995). Previous ERP studies have found a sustained negative slow wave (NSW) over the posterior area during VSTM maintenance, showing increased amplitudes with increased VSTM load (Ruchkin et al., 1992, 1995). Researchers proposed that the increased amplitude of NSW may be associated with either increased representations maintained in VSTM, or with increased processing resources used towards larger memory arrays (Bosch et al., 2001; Vogel and Machizawa, 2004; Zimmer, 2008). Rösler et al. (1997) further found that larger NSW amplitudes were associated with better VSTM performance. In recent years, Vogel et al. (2005) used a bilateral display of stimuli and asked participants to memorize a single hemifield stimulus. The study showed a contralateral and ipsilateral NSW according to the hemifield of the remembered stimulus. Furthermore, the contralateral NSW amplitude increased with increased VSTM load (Vogel et al., 2005; Fukuda et al., 2010). In addition, the frontal cortex is thought to play a key role in cognitive control, responsible for suppressing distractors and maintaining task-relevant information (Jha et al., 2004; Dolcos and McCarthy, 2006; Dolcos et al., 2008). McEvoy et al. (1998) have found a sustained frontal positive slow wave (PSW) during VSTM maintenance, with enhanced amplitudes when both verbal and spatial VSTM load is increased. The authors suggested that these responses were sensitive to high-order attentional demands with frontal areas serving as top-down attentional control for orienting sustained attention to task-relevant representations. Therefore, to measure the late suppression of task-irrelevant distractors, we used the NSW/PSW as the component of interest. We examined whether emotional distractors, containing negative and positive faces, gained unnecessary access to VSTM, and whether this would result in an amplitude increase of the NSW/PSW compared with neutral faces.

In the present study, we manipulated the VSTM load and the valence of emotional distractors to measure the interference of emotion distraction on VSTM performance and neural activity. This work could serve to further clarify aspects related to the temporal dynamics of the interactions between cognitive load and emotional valence, and their relation with the predictions of the “load theory of attention and cognitive control” for high-level processing (Lavie et al., 2004). We employed the method of event-related potentials (ERPs) and divided the ERPs during the maintenance interval into early and late components. The P1/N1 was defined as the early perception and attention of emotional distractors. Hence, we hypothesized that there will be an enhanced P1/N1 activity for negative faces based on the negativity attentional bias. If VSTM load modulates the neural correlates of emotional distraction in an “early” phase, we hypothesized that the increasing VSTM load would decrease this early activity and decline the negativity bias, based on the Lavie’s load theory of attention. The P300 is linked to the demands of “perceptual-central” resources, thus we hypothesized that the increases of VSTM load would result in P300 amplitude decreases. The NSW/PSW was defined as the late sustained attention for task-relevant representations and suppression of emotional distractors, thus our hypothesis was that high VSTM load will increase NSW/PSW amplitudes. If VSTM load modulates the neural correlates of emotional distraction in the “late” phase, we hypothesized that emotional distractors would gain unnecessary access to VSTM in the low-load condition, resulting in decreased P300 amplitudes and increased NSW/PSW amplitudes for emotional faces compared with neutral faces. We expect this effect to be absent in the high-load condition.

To test the above hypotheses, we employed a delayed-match-to-sample (DMS) task using distinctly colored circles (Fukuda et al., 2010) in which encoding and reporting are trivially easy, so that imperfect performance represents the loss of VSTM maintenance. Emotional faces were presented after the encoding phase, for 50 ms which is a shorter duration time compared with previous studies (MacNamara et al., 2012). Participants were required to memorize the colors of one to six circles and ignore the emotional face distractors (Chinese Facial Affective Picture System, CFAPS; Lu et al., 2005) during the maintenance interval. Behavioral data (including accuracy, discrimination index and RTs) and ERP data were recorded and analyzed.

## Materials and Methods

### Participants

Fourteen right-handed undergraduate students (2 female) with normal or corrected-to-normal vision participated in the study. The mean age of the participants was 23.5 years, ranging from 21 to 25 years. One participant’s data was excluded due to technical problems. Another participant’s data was excluded due to excessive blinking and other artifacts. The study was approved by the University of Electronic Science and Technology of China Ethics Board. Written informed consent was signed by each participant in accordance with an experimental protocol approved by the University of Electronic Science and Technology of China Ethics Board before the experiment. The methods were carried out in accordance with the approved guidelines.

### Materials

Seven distinct colored circles (red, green, blue, yellow, cyan, purple, and white) were chosen to measure VSTM capacity. The diameter of each circle was 3.2°. All colored circles were modified using Adobe Photoshop 7.0 (Adobe Systems, San Jose, CA, USA) to achieve uniform luminance, saturation and resolution. 70 positive (35 female), 70 negative (35 female) and 70 neutral faces (35 female) were picked from the Chinese Affective Picture System (Lu et al., 2005). Positive faces were selected from happy faces and negative faces were selected from angry faces. The viewing angle was 9.5° × 11°. All the stimuli were presented with EPRIME software on the monitor screen (32 × 24 cm^2^). Participants sat 60 cm in front of the computer screen.

### Procedure and Task

**Figure 1:**
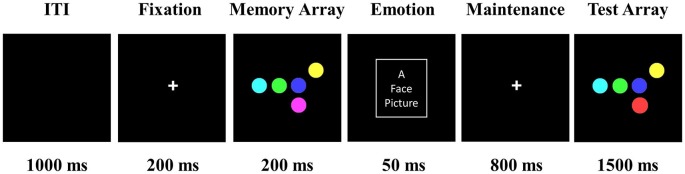
**Trial demonstration of delayed-match-to-sample (DMS) task.** Participants were told to memorize the colors of one to six circles, which were presented for 200 ms (five color circles presented in this figure as an example). Following this, a positive, negative, or neutral face distractor was flashed for 50 ms at the location of the white box (the white box marker was invisible during the experiment). After an 800 ms maintenance interval, participants pressed a button to indicate whether the “Test Array” matched the “Memory Array” or not. The emotional face picture was picked from the Chinese Affective Picture System (Lu et al., 2005).

Figure 1 depicts a sample trial from the DMS task. Each trial began with a fixation (200 ms), during which time participants fixated on a white cross that was centrally presented against a black background. The memory array consisted of 1–6 colored circles, randomly selected without replacement from the set of seven colors, shown randomly in the 3 × 3 matrix (13° × 13° viewing angle) region at the center of black background. The distance between circles was at least 3.8° (center to center). Participants were told to memorize the colors of one to six circles which were presented for 200 ms. At the offset of the memory array, a task-irrelevant emotional face distractor was briefly flashed centrally for 50 ms. We selected 50 ms as the duration of the face distractor in order to let participants avoid doing additional processes of faces, such as passing view, focusing on the facial salient areas, or mental regulation of emotional faces (up- or down-regulate emotion), which might produce different results (Calvo and Beltrán, 2013; Wessing et al., 2013; Calvo et al., 2014). Furthermore, a long presentation duration of a face distractor during the VSTM maintenance interval would attenuate the memory of previous task-relevant information, no matter what kind of emotional distractors are used. Previous studies have shown that happy and angry faces can be discriminated during the 50 ms presentation (Grimshaw et al., 2004). ERP studies used 50 ms to measure the early attention and fast detection of emotional faces, resulting in different P1 and N1 components for different emotional faces (Dennis and Chen, 2007; Eimer et al., 2008). In the present study, we examined how the VSTM load modulates the early perception of face distractors using P1 and N1 components. Participants were instructed to ignore the emotional face. After the maintenance interval of 800 ms, participants pressed a button to indicate whether the “Test Array” matched the “Memory Array” or not. Participants were told to respond using only one finger, pressing 1 for a match and 2 for no match, as quickly and accurately as possible. The color of one circle in the test array was different from the corresponding item in the memory array in 50% of the trials.

Each participant performed two practice blocks and 20 test blocks. Each block included 72 trials (a total of 1440 trials). Our experimental design was a 3 × 6 factorial with the within-subject factors being emotion (positive, negative and neutral) and VSTM load (one, two, three, four, five and six). Trial order was varied randomly within each block for each participant.

### Data Recording and Processing

Participants were seated comfortably in front of the computer in a dark and silent room. Electroencephalography (EEG) signals were collected using a 128-channel EGI HydroCel GSN (EGI, Eugene, OR, USA). EEG data were sampled at 1000 Hz with an amplifier band-pass of 0.1–48 Hz and recorded using NetStation 4.1.2 (EGI Software: EGI, Eugene, OR, USA). Electrode impedances were kept under 50 KΩ at the beginning of the session. All channels were referenced to Cz (129th) during recording and re-referenced to an average reference off-line. EEG data were processed off-line using Net Station Waveform Tools: 1. Filter: An FIR 0.1–30 Hz bandpass filter was applied; 2. Segmentation: the data were segmented from 200 ms before the onset of the memory array to 1600 ms after the memory array onset; 3. Artifact Detection and Bad Channel Replacement: for each segments, channels with amplitude exceeded 200 μV were marked as bad and replaced through the interpolation of neighboring electrodes; and 4. File Export: all of the data were exported in .mat format (data from one participant was excluded due to technical problems). MATLAB software was used to exclude the bad segments which contained significant eye movements, blinks, muscle artifact (potential exceeding ± 100 μV), or contained an incorrect button press. We decomposed the data into 40 ICs using the PCA method (EEGLAB toolbox). According to the scalp maps and time-courses, we identified and removed the eye movement artifact during the maintenance interval. This rejection retained at least 84% of original trials for each condition. One participant’s data was excluded due to excessive blinking and other artifacts. The remaining segments were baseline corrected using the 200 ms before the onset of the memory array. Segments were then averaged across trials according to load (one to six) and emotional faces (positive, negative and neutral).

### Behavioral Analysis

We calculated the VSTM capacity using the equation: *K* = *S* × (Hit rate − False alarm rate)/(1 − False alarm rate; Pashler, 1988), where S is the load (the number of color circles in the memory array), the hit rate is the conditional probability that participants accurately reported matches, and the false alarm rate is the conditional probability that participants reported a match when the two arrays did not match. It was easy for subjects to memorize one or two items (accuracy: 94 ± 1% for positive, 93 ± 1% for negative and 94 ± 0.8% for neutral face). Therefore we split the data for subsequent analyses, such that data with loads one and two were averaged into a low-load condition and data with loads 3–6 were averaged into a high-load condition. For the low-load condition, 1460, 1422 and 1445 trials remained in total across all subjects for positive, negative and neutral faces, respectively. For the high-load condition, 2374, 2281 and 2317 trials remained in total across all subjects for positive, negative and neutral faces, respectively. To balance the trial number of low load and high load conditions, we selected the trials with equal numbers between low load and high load for each distractor, for each subject randomly. An average of 121 (sd 24), 118 (sd 25), 120 (sd 25) trials were left for each face distractors.

We calculated the accuracy and mean RTs across all participants by using a 2 × 3 repeated measure analysis of variance (ANOVA) with independent repeated factors load (low-load and high-load) and emotional faces (positive, negative and neutral). Only RTs less than 1200 ms were included for accuracy analysis, and only correct responses were applied to measure RTs. In addition, discrimination index was defined as: d′ = Z (hit rate) − Z (false alarm rate), where Z (P) is the z-score associated with probability P by the standard normal distribution.

### ERP Analysis

ERPs were measured for the following regions of interest (ROIs): frontal (F3, FZ and F4), central (C3, CZ and C4), parietal (P3, PZ and P4) and occipital (PO7, OZ and PO8) regions. We defined the first peak during the maintenance interval as the early component, with the P1 at occipital sites (PO7, OZ and PO8) and the N1 at frontal sites (F3, FZ and F4). The peak values and corresponding latency of P1 and N1 in the 70–200 ms time-window post face onset were used in subsequent statistical analysis. The P300 was measured using the mean amplitude in the 300–500 ms time-window at parietal sites (P3, PZ and P4). Between 600–1050 ms, the late components were defined as the NSW at occipital sites and the PSW at frontal sites. The mean amplitudes for the 600–1050 ms time-window were used in subsequent statistical analysis.

### Regression Analysis

Linear regression models were used to evaluate the relationship between behavioral performance and ERP activity. Since it was easy for subjects to memorize one or two items (accuracy: 94% for positive, 93% for negative and 94% for neutral face), the ERP activity for the low-load condition served as the baseline and the difference score between high and low load was measured. The difference score of P1/N1 amplitude and latency was used to correlate with RT costs (high-load minus low-load) separately.

### Statistical Analysis

Statistical analysis was performed using SPSS Statistics Release 19 (IBM, Somers, NY, USA) General Linear Model. Bonferroni corrections were performed for multiple comparisons and Greenhouse-Geisser epsilon corrections were performed for non-sphericity data where necessary. All regression analyses used Pearson’s linear correlation coefficient.

## Results

### Behavioral Results

**Figure 2:**
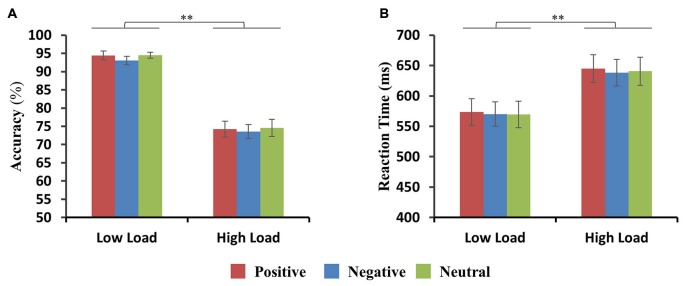
**Behavioral results. (A,B)** Accuracy and reaction times (RTs) are shown for positive, negative and neutral faces during the low-load and high-load condition. Error bars represent the standard error of the mean (SEM) (**p < 0.01).

Accuracy (percent correct) and RTs for positive, negative and neutral faces for low-load and high-load conditions are shown in Figures 2A,B. For accuracy, there was a significant main effect of load (*F*_(1,11)_ = 188.4, *p* < 0.001) with higher accuracy in the low-load than in the high-load condition. Neither main effect of emotion nor interactions between VSTM load and emotion were found. For RTs, there was a significant main effect of load (*F*_(1,11)_ = 59.9, *p* < 0.001) with longer RTs in the high-load than the low-load condition. No main effect of emotion and no interactions between VSTM load and emotion were found. Two-way repeated ANOVA of accuracy and RTs with emotion (positive, negative, and neutral) and load (1, 2, 3, 4, 5, 6) as factors revealed a main effect of load (ACC: *F*_(5,55)_ = 61.5, *p* < 0.001; RTs: *F*_(5,55)_ = 25.1, *p* < 0.001), but there was no significant main effect for emotion nor a significant interaction. Similarly, there were no significant differences in maximum VSTM capacity for positive (4.6 ± 0.28 items), negative (4.8 ± 0.29 items) and neutral (4.4 ± 0.27 items) distraction (ACC: *F*_(2,11)_ = 1.32, *p* = 0.3).

We also calculated the discrimination index (d′) for positive, negative and neutral faces for low-load and high-load conditions. This revealed only a significant main effect of VSTM load (*F*_(1,11)_ = 205, *p* < 0.001) with higher discrimination index in the low-load than high-load condition.

### ERP Results

**Figure 3:**
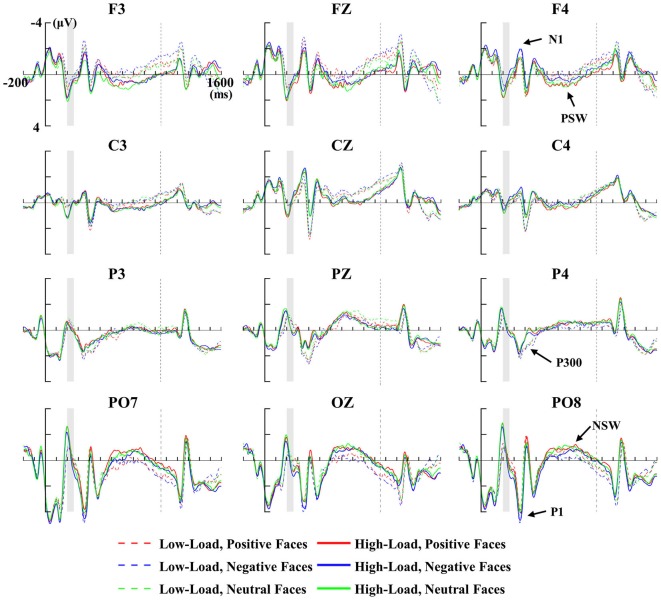
**Memory-locked ERPs for positive, negative and neutral faces during the low-load (dashed line) and high-load (solid line) at ROI electrodes.** The vertical thick line indicates the onset of memory array, and the shadowed period indicates the presentation of face distractors, and the vertical dashed line indicates the onset of the test array. Face-related P1/N1, P300, positive slow wave (PSW) and negative slow wave (NSW) are marked in the figure.

Figure 3 depicts the grand-averaged ERP waveforms of positive, negative and neutral faces for low and high loads at the ROIs: frontal (F3, FZ and F4), central (C3, CZ and C4), parietal (P3, PZ and P4) and occipital (PO7, OZ and PO8) regions. Emotional face distractors were associated with a relative negative shift over frontal sensors and a relative positive shift over occipital sensors around 150 ms post face onset. An overall 2 × 3 × 2 × 3 ANOVA with factors of site (frontal and occipital), hemisphere (left, middle and right), load (low-load and high-load) and emotional faces (positive, negative and neutral) revealed a significant interaction of site × emotion (*F*_(2,10)_ = 5.96, *p* < 0.05), a significant interaction of hemisphere × load (*F*_(2,10)_ = 4.87, *p* < 0.05), and a significant main effect of hemisphere (*F*_(2,10)_ = 7.13, *p* < 0.05) with bigger amplitudes for lateral vs. middle electrode sites. Then we performed a 3 (left, middle and right) × 2 (low-load and high-load) × 3 (positive, negative and neutral) for frontal N1 and occipital P1 separately, as shown below.

For the late sustained slow wave, VSTM load was associated with a relative positive shift slow wave over frontal sensors and a relative negative shift slow wave over occipital sensors. A 2 × 3 × 2 × 3 repeated ANOVA with site (frontal and occipital), hemisphere (left, middle and right), load (low-load and high-load) and emotional faces (positive, negative and neutral) revealed a significant interaction of site × load (*F*_(1,11)_ = 6.41, *p* < 0.05). Hence, we performed a 3 (left, middle and right) × 2 (low-load and high-load) × 3 (positive, negative and neutral) repeated ANOVAs for frontal PSW and occipital NSW separately, as shown below.

#### P1 Component

**Figure 4:**
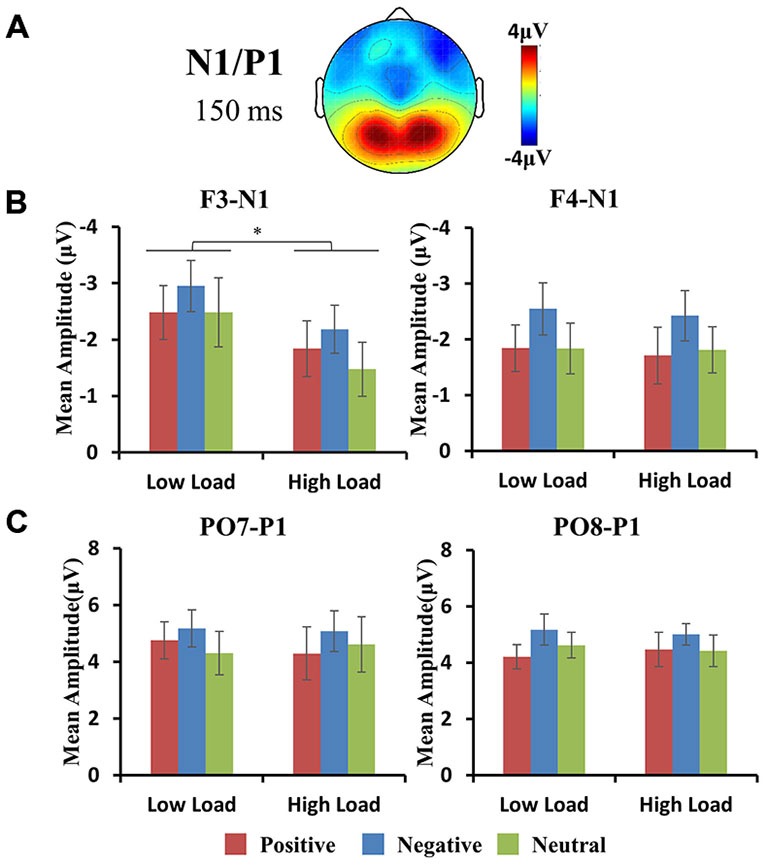
**(A)** Topographic map of face-related N1/P1 component. Histograms show the peak amplitudes of N1 at frontal (**B** row) and P1 at occipital (**C** row) sites. Error bars represent the SEM (*p < 0.05). Negative faces elicited larger N1 amplitudes vs. positive and neutral faces, and larger P1 amplitudes vs. positive faces.

The maximum amplitudes for the P1 were observed at occipital regions (Figures 3, 4A,C). A repeated 3 × 2 × 3 (hemisphere × load × emotion) ANOVA of P1 amplitudes revealed a significant main effect of emotion (*F*_(2,10)_ = 5.39, *p* < 0.05). *Post hoc* *t*-tests revealed higher amplitude for negative vs. positive face distractors (*t*_(11)_ = 3.44, *p* < 0.05). For the latency of P1, an identical ANOVA revealed a marginally significant main effect of load (*F*_(1,11)_ = 3.4, *p* = 0.09) with delayed latency for high vs. low loads, and a significant main effect of emotion (*F*_(2,10)_ = 4.99, *p* < 0.05). *Post hoc* *t*-test revealed delayed latency for negative vs. neutral faces (*t*_(11)_ = 3.12, *p* < 0.05) and positive vs. neutral faces (*t*_(11)_ = 2.78, *p* = 0.05) separately. There were no interactions for P1 amplitude and latency.

#### N1 Component

The maximum amplitudes for the N1 were observed at frontal regions (Figures 3, 4A,B). A repeated 3 × 2 × 3 (hemisphere × load × emotion) ANOVA of P1 amplitudes revealed a significant interaction of hemisphere × load (*F*_(2,10)_ = 4.33, *p* < 0.05), a significant main effect of emotion (*F*_(2,10)_ = 4.36, *p* < 0.05), and a significant main effect of load (*F*_(1,11)_ = 6.89, *p* < 0.05). Hence, we performed a 2 × 3 (load × emotion) repeated ANOVAs for each frontal hemisphere separately. The amplitude of N1 showed a main effect of emotion at F3 and F4 (F3: *F*_(2,22)_ = 5.9, *p* < 0.05; FZ: *F*_(2,22)_ = 1.73, *p* = 0.23; F4: *F*_(2,22)_ = 9.4, *p* < 0.01) with higher amplitude for negative compared with other face distractors, and a main effect of load at F3 (*F*_(1,11)_ = 12.1, *p* < 0.01) with higher amplitude in low-load than high-load conditions, but no interaction was found. There were no main effects or interactions for N1 latency at frontal regions.

#### P300 Component

**Figure 5:**
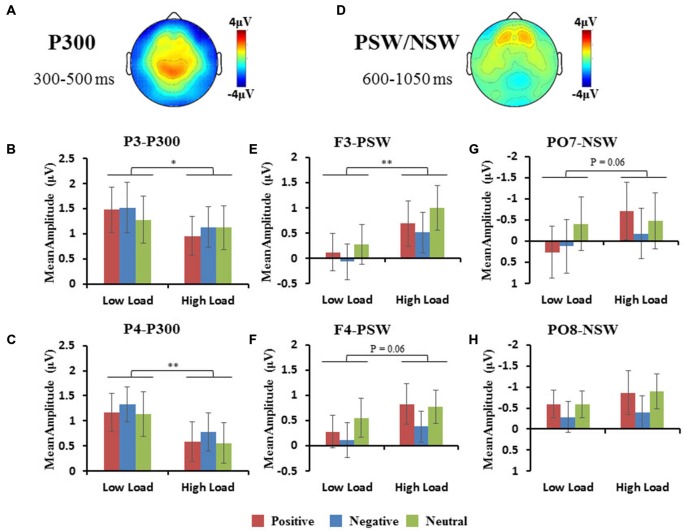
**(A,D)** Topographic map of P300 and PSW/NSW component. Histograms show the mean amplitudes of P300 at P3 and P4 **(B,C)**, the mean amplitudes of frontal PSW **(E,F)**, and the mean amplitudes of occipital NSW **(G,H)**. Error bars represent the SEM (*p < 0.05, **p < 0.01).

The area of the P300 component was larger at parietal regions (Figure 5A). A repeated 3 × 2 × 3 (hemisphere × load × emotion) ANOVA of P300 amplitudes revealed a significant interaction of hemisphere × load (*F*_(2,10)_ = 6.47, *p* < 0.05), a significant main effect of load (*F*_(1,11)_ = 13, *p* < 0.01). Hence, we performed a 2 × 3 (load × emotion) repeated ANOVAs for each parietal hemisphere separately. Two-way repeated ANOVA of P300 amplitude revealed a main effect of load (P3: *F*_(1,11)_ = 6.27, *p* < 0.05; PZ: *F*_(1,11)_ = 15.25, *p* < 0.01; P4: *F*_(1,11)_ = 11.65, *p* < 0.01), with higher amplitudes in the low-load compared with the high-load condition (Figures 5B,C). We did not observe a main effect of emotion nor an interaction.

#### PSW and NSW Components

The area of the PSW components were observed at frontal regions (Figures 3, 5E,F), and NSW components were observed at occipital regions (Figures 3, 5G,H). A repeated 3 × 2 × 3 (hemisphere × load × emotion) ANOVA of frontal PSW amplitudes revealed a significant main effect of load (*F*_(1,11)_ = 13, *p* < 0.01), with higher amplitude for high vs. low loads. Identical ANOVA of occipital NSW amplitudes revealed a significant main effect of load (*F*_(1,11)_ = 5.54, *p* < 0.05), with higher amplitude for high vs. low loads. We did not observe a main effect of emotion nor an interaction.

### Correlations Between ERP and Behavior

**Figure 6:**
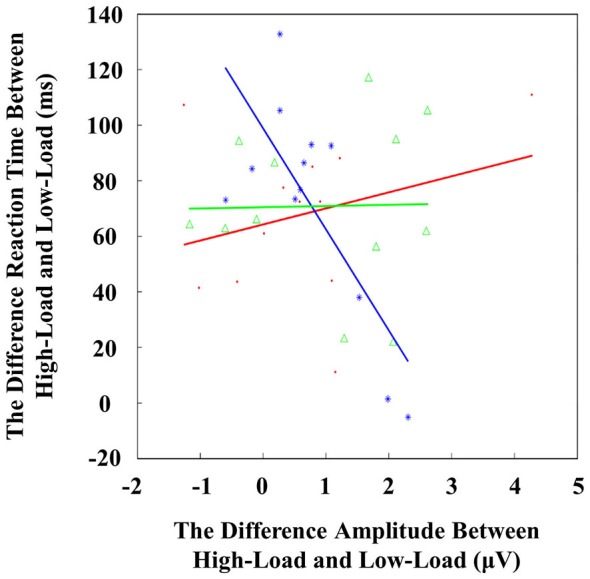
**Regression between the visual short-term memory (VSTM) load-related change in N1 amplitude (High-Load minus Low-Load) and the VSTM load-related change in RT (High-Load minus Low-Load) for positive face (red), negative face (blue) and neutral face (green) conditions, respectively.** Pearson correlation coefficient (*r* = −0.759) was significant (*p* = 0.004) for negative faces only.

We computed a correlation analysis to measure the relationships between the early ERP activity and VSTM performance for three types of distractors, respectively. We found an inverse correlation between the N1 activation difference (high-load minus low-load) and RT costs (high-load minus low-load) when viewing negative face distractors at electrode site F3 (*r* = −0.76, *p* < 0.01; Figure 6). There was no correlation for the positive (*r* = 0.28, *p* = 0.38) and neutral (*r* = 0.02, *p* = 0.95) distractor conditions. In addition, there was no significant correlation between P1 latency changes and RT costs (all *r* < 0.4 and all *p* > 0.2).

## Discussion

Due to the limited capacity of VSTM, more resources (including attentional and processing resources) are allocated to task-relevant information, resulting in fewer resources allocated to distractors and less distractibility. The aim of the present study was to investigate the time course of emotional distraction on maintenance of task-relevant information in VSTM, and the modulation of such interference by VSTM load.

In line with previous work, negative face distractors elicited larger N1 amplitudes compared with positive and neutral distractors, which were maximal at frontal sites (Eimer and Holmes, 2002; Luo et al., 2010). Wenbo suggested that this early frontal N1 represented the rapid detection of facial expression in the frontal cortex, especially for negative faces. Such negativity bias is valuable for recognizing threatening information in the real world. Eimer et al. suggested that frontal N1 represented the rapid attentional alerting to emotional faces (Eimer et al., 2008). Negative faces preferentially attract attention early in the information processing stream, as reflected by larger N1 amplitudes (Olofsson et al., 2008). In addition, the frontal N1 amplitude was reduced by VSTM load, which may indicate that the high VSTM load exhausted limited attentional resources, resulting in less attention allocated to distractors (Lavie et al., 2004). An alternative interpretation could be that high cognitive load demands enhances top-down control, which in turn acts to inhibit emotional processing. In the current study, an inverse correlation was observed between the N1 activation difference (high-load minus low-load) and RTs difference (high-load minus low-load) when viewing negative face distractors at the F3 electrode (Figure 6), indicating that individuals with larger decreases in N1 activity showed smaller RT costs from low-load to high-load, and* vice versa*. This finding suggests that for the high-load condition, when there is less early attention allocation to negative distractors, VSTM performance improves, since RTs in the high-load condition are almost as fast as the RTs in the low-load condition. However, there was no significant correlation between N1 activation difference and RTs difference in the positive or neutral distractor condition. This finding suggests that frontal areas may be involved in resisting emotional interference in the early phase (Jha et al., 2004; Anticevic et al., 2010; Ziaei et al., 2014).

As expected, we found larger P1 activity in response to negative compared with positive distractors. Consistent with our findings, P1 amplitudes for fear or angry faces at occipital sites have been shown to be larger than those for positive or neutral faces (Batty and Taylor, 2003; Rellecke et al., 2012), suggesting a rapid and automatic emotional processing. Moreover, some researchers found such an effect in the brief presentation of faces and suggested it reflected a rapid extraction of emotional information before fine-grained perceptual processing begins (Pourtois et al., 2004; Dennis and Chen, 2007). In addition, previous studies have reported that P1 latency for faces that need to be ignored were significantly longer than attended faces, suggesting P1 was modulated by top-down attention control (Clapp et al., 2010). In our study, we found significant delayed P1 latency for negative vs. neutral distractors. As the P1 latency shift reflects the large neurons engaged in visual association cortex (VAC; Lopes da Silva, 1991), this may suggest that ignoring negative faces is harder than ignoring neutral faces and thus more processing resources are required. Moreover, we found that high VSTM load prolongs P1 latency when compared with low VSTM load, suggesting that neural processing speed of ignored distractors was slowed down. This finding may imply that the action of ignoring distractors becomes more difficult and ineffective since the VSTM capacity is insufficient. Previous studies, using inverse and upright faces as stimuli, found P1 latency to be delayed, suggesting that inverse faces undermine the holistic processing of a face (Itier and Taylor, 2002, 2004; Meeren et al., 2005). Thus, an alternative interpretation of the delayed P1 latency observed in the current study for the high-load condition could be that high VSTM load disrupts the early facial expression processing of a face.

Researchers have proposed that the P300 amplitude reflects demands on “perceptual-central” resources or processing capacity (Kramer and Spinks, 1991). Previous studies have shown that P300 over parietal regions is related to VSTM load, and that amplitude decreases with increasing VSTM load (Kok, 2001; Watter et al., 2001; Busch and Herrmann, 2003). Consistent with above studies, we found a decreased amplitude of P300 from low-load to high-load. This finding suggests that increasing VSTM load attenuates the perceptual-central resources and consumes the limited VSTM capacity, as reflected by decreased P300 amplitudes (Kok, 2001).

Late slow wave components were used to measure the late attentional control engaged in representational selection between task-relevant information and distractors. The current results revealed that VSTM load increased occipital NSW activity. This result is in line with previous studies reporting that the sustained activity during VSTM maintenance is related to the amount of information retained in VSTM (Ruchkin et al., 1992, 1995). Studies using the bilateral stimulus, have found that the contralateral NSW amplitude was strongly modulated by the amount of color objects held in VSTM (Vogel et al., 2005; Fukuda et al., 2010).

Studies have shown that the frontal cortex is associated with VSTM maintenance and manipulation, and that it plays a role in allocating attention toward or away from information, via top-down communication with sensory cortices (Curtis and D’Esposito, 2003; Dolcos and McCarthy, 2006; Ranganath, 2006). McEvoy et al. (1998) have found that the frontal slow wave activity was enhanced with the increasing verbal and spatial VSTM load, suggesting that these responses were sensitive to high-order attentional demands in the task. Similar results have also been found in auditory VSTM tasks (Monfort and Pouthas, 2003). The present results revealed that the frontal PSW activity was higher in the high-load condition than in the low-load condition, which may be due to the greater working memory or attentional demands associated with the high-load condition.

Our results showed no effect of emotional faces on the frontal PSW and occipital NSW. One possibility is that the very brief presentation duration of the face (50 ms) in the present study decreased the salience of emotional faces, thus having no impact on task-relevant information maintained in VSTM, which is in line with behavioral results. Another possibility is that the brief duration of the faces prevents the participants from further processing of faces, including response selection and decision (Calvo et al., 2013).

In summary, we found that high VSTM load modulates the early perception of emotional distractors (P1, N1). This is in line with Lavie et al.’s (2004) early selective attention theory, showing that perception of irrelevant distractors is reduced during a high load condition, suggesting insufficient capacity for distractor processing. Moreover, individuals with larger decreases in N1 activity showed smaller RT costs from low-load to high-load when viewing negative face distractors. However, emotional distractors had no effect on the late VSTM-related activities (P300, PSW and NSW) and VSTM behavioral performance. This may be due to the short duration of presentation of faces, which were easily ignored and thus subjects did not need further late cognitive control suppression.

## Conclusion

The present study illustrates the modulation effects of VSTM on emotional processes. Negative faces elicited higher frontal N1 and occipital P1 activity, reflecting rapid attentional alerting and early perceptual processing of negative faces. In addition, increasing VSTM load reduced the N1 activity and delayed the P1 latency. These results are in line with Lavie et al.’s (2004) early selection view that perception of irrelevant distractors is reduced during the high load condition due to the insufficient capacity for distractor processing. An alternative interpretation could be that high cognitive load demands enhanced top-down control, which in turn acts to inhibit emotional processing. The late P300 response decreased with increasing VSTM load, suggesting that increasing VSTM load attenuates the perceptual-central resources and consumes the limited VSTM capacity. The late frontal PSW and occipital NSW responses were sensitive to increases in VSTM load, suggesting higher VSTM or attentional demands during the high-load vs. the low-load condition. Emotional interference effect was not found in the late VSTM-related P300, PSW and NSW components. The present findings support the evidence that VSTM modulates emotional distraction in the early phase. Longer duration of presentation of face distractors may be helpful in further understanding how VSTM modulates emotional distraction in the late phase.

## Conflict of Interest Statement

The authors declare that the research was conducted in the absence of any commercial or financial relationships that could be construed as a potential conflict of interest.

## References

[B1] Anticevic A. Repovs G. Barch D. M. (2010). Resisting emotional interference: brain regions facilitating working memory performance during negative distraction. Cogn. Affect. Behav. Neurosci. 10, 159–173. 10.3758/cabn.10.2.159 20498341 PMC3856369

[B2] Baddeley A. (2003). Working memory: looking back and looking forward. Nat. Rev. Neurosci. 4, 829–839. 10.1038/nrn1201 14523382

[B3] Batty M. Taylor M. J. (2003). Early processing of the six basic facial emotional expressions. Brain Res. Cogn. Brain Res. 17, 613–620. 10.1016/s0926-6410(03)00174-5 14561449

[B4] Bosch V. Mecklinger A. Friederici A. D. (2001). Slow cortical potentials during retention of object, spatial and verbal information. Brain Res. Cogn. Brain Res. 10, 219–237. 10.1016/s0926-6410(00)00040-9 11167047

[B5] Busch N. A. Herrmann C. S. (2003). Object-load and feature-load modulate EEG in a short-term memory task. Neuroreport 14, 1721–1724. 10.1097/01.wnr.0000087727.58565.1b 14512845

[B6] Calvo M. G. Beltrán D. (2013). Recognition advantage of happy faces: tracing the neurocognitive processes. Neuropsychologia 51, 2051–2060. 10.1016/j.neuropsychologia.2013.07.010 23880097

[B7] Calvo M. G. Beltrán D. Fernández-Martín A. (2014). Processing of facial expressions in peripheral vision: neurophysiological evidence. Biol. Psychol. 100, 60–70. 10.1016/j.biopsycho.2014.05.007 24880051

[B8] Calvo M. G. Marrero H. Beltrán D. (2013). When does the brain distinguish between genuine and ambiguous smiles? An ERP study. Brain Cogn. 81, 237–246. 10.1016/j.bandc.2012.10.009 23262178

[B9] Chafee M. V. Goldman-Rakic P. S. (2000). Inactivation of parietal and prefrontal cortex reveals interdependence of neural activity during memory-guided saccades. J. Neurophysiol. 83, 1550–1566. 10712479 10.1152/jn.2000.83.3.1550

[B10] Clapp W. C. Rubens M. T. Gazzaley A. (2010). Mechanisms of working memory disruption by external interference. Cereb. Cortex 20, 859–872. 10.1093/cercor/bhp150 19648173 PMC2837090

[B11] Clark V. P. Hillyard S. A. (1996). Spatial selective attention affects early extrastriate but not striate components of the visual evoked potential. J. Cogn. Neurosci. 8, 387–402. 10.1162/jocn.1996.8.5.387 23961943

[B12] Clarke R. Johnstone T. (2013). Prefrontal inhibition of threat processing reduces working memory interference. Front. Hum. Neurosci. 7:228. 10.3389/fnhum.2013.00228 23750133 PMC3667546

[B13] Curtis C. E. D’Esposito M. (2003). Persistent activity in the prefrontal cortex during working memory. Trends Cogn. Sci. 7, 415–423. 10.1016/s1364-6613(03)00197-9 12963473

[B14] Dennis T. A. Chen C. C. (2007). Emotional face processing and attention performance in three domains: neurophysiological mechanisms and moderating effects of trait anxiety. Int. J. Psychophysiol. 65, 10–19. 10.1016/j.ijpsycho.2007.02.006 17383040 PMC2001230

[B15] Di Russo F. Martínez A. Sereno M. I. Pitzalis S. Hillyard S. A. (2002). Cortical sources of the early components of the visual evoked potential. Hum. Brain Mapp. 15, 95–111. 10.1002/hbm.10010 11835601 PMC6871868

[B16] Dolcos F. McCarthy G. (2006). Brain systems mediating cognitive interference by emotional distraction. J. Neurosci. 26, 2072–2079. 10.1523/jneurosci.5042-05.2006 16481440 PMC6674921

[B17] Dolcos F. Diaz-Granados P. Wang L. McCarthy G. (2008). Opposing influences of emotional and non-emotional distracters upon sustained prefrontal cortex activity during a delayed-response working memory task. Neuropsychologia 46, 326–335. 10.1016/j.neuropsychologia.2007.07.010 17765933

[B18] Donchin E. Coles M. G. H. (1988). Is the P300 component a manifestation of context updating? Behav. Brain Sci. 11, 357–374. 10.1017/s0140525x00058027

[B19] Eimer M. Holmes A. (2002). An ERP study on the time course of emotional face processing. Neuroreport 13, 427–431. 10.1097/00001756-200203250-00013 11930154

[B20] Eimer M. Kiss M. Holmes A. (2008). Links between rapid ERP responses to fearful faces and conscious awareness. J. Neuropsychol. 2, 165–181. 10.1348/174866407x245411 19330049 PMC2661068

[B21] Erk S. Kleczar A. Walter H. (2007). Valence-specific regulation effects in a working memory task with emotional context. Neuroimage 37, 623–632. 10.1016/j.neuroimage.2007.05.006 17570686

[B22] Fukuda K. Awh E. Vogel E. K. (2010). Discrete capacity limits in visual working memory. Curr. Opin. Neurobiol. 20, 177–182. 10.1016/j.conb.2010.03.005 20362427 PMC3019116

[B23] Gazzaley A. (2011). Influence of early attentional modulation on working memory. Neuropsychologia 49, 1410–1424. 10.1016/j.neuropsychologia.2010.12.022 21184764 PMC3086962

[B24] Gazzaley A. Clapp W. Kelley J. McEvoy K. Knight R. T. D’Esposito M. (2008). Age-related top-down suppression deficit in the early stages of cortical visual memory processing. Proc. Natl. Acad. Sci. U S A 105, 13122–13126. 10.1073/pnas.0806074105 18765818 PMC2529045

[B25] Gazzaley A. Cooney J. W. McEvoy K. Knight R. T. D’Esposito M. (2005). Top-down enhancement and suppression of the magnitude and speed of neural activity. J. Cogn. Neurosci. 17, 507–517. 10.1162/0898929053279522 15814009

[B26] Grimshaw G. M. Bulman-Fleming M. B. Ngo C. (2004). A signal-detection analysis of sex differences in the perception of emotional faces. Brain Cogn. 54, 248–250. 10.1016/j.bandc.2004.02.029 15050785

[B27] Hillyard S. A. Vogel E. K. Luck S. J. (1998). Sensory gain control (amplification) as a mechanism of selective attention: electrophysiological and neuroimaging evidence. Philos. Trans. R. Soc. Lond. B Biol. Sci. 353, 1257–1270. 10.1098/rstb.1998.0281 9770220 PMC1692341

[B28] Holmes A. Vuilleumier P. Eimer M. (2003). The processing of emotional facial expression is gated by spatial attention: evidence from event-related brain potentials. Brain Res. Cogn. Brain Res. 16, 174–184. 10.1016/s0926-6410(02)00268-9 12668225

[B29] Iordan A. D. Dolcos S. Dolcos F. (2013). Neural signatures of the response to emotional distraction: a review of evidence from brain imaging investigations. Front. Hum. Neurosci. 7:200. 10.3389/fnhum.2013.00200 23761741 PMC3672684

[B30] Itier R. J. Taylor M. J. (2002). Inversion and contrast polarity reversal affect both encoding and recognition processes of unfamiliar faces: a repetition study using ERPs. Neuroimage 15, 353–372. 10.1006/nimg.2001.0982 11798271

[B31] Itier R. J. Taylor M. J. (2004). Effects of repetition learning on upright, inverted and contrast-reversed face processing using ERPs. Neuroimage 21, 1518–1532. 10.1016/j.neuroimage.2003.12.016 15050576

[B32] Jha A. P. Fabian S. A. Aguirre G. K. (2004). The role of prefrontal cortex in resolving distractor interference. Cogn. Affect. Behav. Neurosci. 4, 517–527. 10.3758/cabn.4.4.517 15849894

[B33] Kim S.-Y. Kim M.-S. Chun M. M. (2005). Concurrent working memory load can reduce distraction. Proc. Natl. Acad. Sci. U S A 102, 16524–16529. 10.1073/pnas.0505454102 16258067 PMC1283430

[B34] Kok A. (2001). On the utility of P3 amplitude as a measure of processing capacity. Psychophysiology 38, 557–577. 10.1017/s0048577201990559 11352145

[B35] Kong X. Chen X. Tan B. Zhao D. Jin Z. Li L. (2013). Sad facial cues inhibit temporal attention: evidence from an event-related potential study. Neuroreport 24, 476–481. 10.1097/wnr.0b013e328361c720 23628713

[B36] Konstantinou N. Lavie N. (2013). Dissociable roles of different types of working memory load in visual detection. J. Exp. Psychol. Hum. Percept. Perform. 39, 919–924. 10.1037/a0033037 23713796 PMC3725889

[B37] Kramer A. Spinks J. (1991). Capacity Views of Human Information Processing. Available online at: http://ezproxy.net.ucf.edu/login?url=http://search.ebscohost.com/login.aspx?direct=true&db=ega&AN=ega126393&site=ehost-live

[B38] Lavie N. Hirst A. de Fockert J. W. Viding E. (2004). Load theory of selective attention and cognitive control. J. Exp. Psychol. Gen. 133, 339–354. 10.1037/0096-3445.133.3.339 15355143

[B39] Lopes da Silva F. (1991). Neural mechanisms underlying brain waves: from neural membranes to networks. Electroencephalogr. Clin. Neurophysiol. 79, 81–93. 10.1016/0013-4694(91)90044-5 1713832

[B70] Luo W. Feng W. He W. Wang N. Y. Luo Y. J. (2010). Three stages of facial expression processing: ERP study with rapid serial visual presentation. Neuroimage 49, 1857–1867. 10.1016/j.neuroimage.2009.09.018 19770052 PMC3794431

[B40] Lu B. Hui M. Yu-Xia H. (2005). The development of native chinese affective picture system–A pretest in 46 college students. Chinese Ment. Heal. J. 19, 719–722.

[B41] MacNamara A. Schmidt J. Zelinsky G. J. Hajcak G. (2012). Electrocortical and ocular indices of attention to fearful and neutral faces presented under high and low working memory load. Biol. Psychol. 91, 349–356. 10.1016/j.biopsycho.2012.08.005 22951516

[B42] McEvoy L. K. Smith M. E. Gevins A. (1998). Dynamic cortical networks of verbal and spatial working memory: effects of memory load and task practice. Cereb. Cortex 8, 563–574. 10.1093/cercor/8.7.563 9823478

[B43] McNab F. Dolan R. J. (2014). Dissociating distractor-filtering at encoding and during maintenance. J. Exp. Psychol. Hum. Percept. Perform. 40, 960–967. 10.1037/a0036013 24512609 PMC4035130

[B44] Meeren H. K. M. van Heijnsbergen C. C. R. J. de Gelder B. (2005). Rapid perceptual integration of facial expression and emotional body language. Proc. Natl. Acad. Sci. U S A 102, 16518–16523. 10.1073/pnas.0507650102 16260734 PMC1283446

[B45] Miendlarzewska E. A. van Elswijk G. Cannistraci C. V. van Ee R. (2013). Working memory load attenuates emotional enhancement in recognition memory. Front. Psychol. 4:112. 10.3389/fpsyg.2013.00112 23515565 PMC3600573

[B46] Monfort V. Pouthas V. (2003). Effects of working memory demands on frontal slow waves in time-interval reproduction tasks in humans. Neurosci. Lett. 343, 195–199. 10.1016/s0304-3940(03)00385-9 12770695

[B47] Norman D. Shallice T. (1986). Attention to action: willed and automatic control of behaviour. Conscious. Self Regul. Adv. Theory Res. 4, 1–18.

[B48] Olofsson J. K. Nordin S. Sequeira H. Polich J. (2008). Affective picture processing: an integrative review of ERP findings. Biol. Psychol. 77, 247–265. 10.1016/j.biopsycho.2007.11.006 18164800 PMC2443061

[B49] Palermo R. Rhodes G. (2007). Are you always on my mind? A review of how face perception and attention interact. Neuropsychologia 45, 75–92. 10.1016/j.neuropsychologia.2006.04.025 16797607

[B50] Pashler H. (1988). Familiarity and visual change detection. Percept. Psychophys. 44, 369–378. 10.3758/bf03210419 3226885

[B51] Pessoa L. (2008). On the relationship between emotion and cognition. Nat. Rev. Neurosci. 9, 148–158. 10.1038/nrn2317 18209732

[B52] Petroni A. Canales-Johnson A. Urquina H. Guex R. Hurtado E. Blenkmann A.. (2011). The cortical processing of facial emotional expression is associated with social cognition skills and executive functioning: a preliminary study. Neurosci. Lett. 505, 41–46. 10.1016/j.neulet.2011.09.062 22001365

[B54] Pourtois G. Grandjean D. Sander D. Vuilleumier P. (2004). Electrophysiological correlates of rapid spatial orienting towards fearful faces. Cereb. Cortex 14, 619–633. 10.1093/cercor/bhh023 15054077

[B55] Ranganath C. (2006). Working memory for visual objects: complementary roles of inferior temporal, medial temporal and prefrontal cortex. Neuroscience 139, 277–289. 10.1016/j.neuroscience.2005.06.092 16343785

[B56] Rellecke J. Sommer W. Schacht A. (2012). Does processing of emotional facial expressions depend on intention? Time-resolved evidence from event-related brain potentials. Biol. Psychol. 90, 23–32. 10.1016/j.biopsycho.2012.02.002 22361274

[B57] Rippon G. Rolls E. Davidson R. D. (2001). The brain and emotion. J. Psychophysiol. 15, 208–210. 10.1027/0269-8803.15.3.208

[B58] Rösler F. Heil M. Röder B. (1997). Slow negative brain potentials as reflections of specific modular resources of cognition. Biol. Psychol. 45, 109–141. 10.1016/s0301-0511(96)05225-8 9083647

[B59] Ruchkin D. S. Canoune H. L. Johnson R. Ritter W. (1995). Working memory and preparation elicit different patterns of slow wave event-related brain potentials. Psychophysiology 32, 399–410. 10.1111/j.1469-8986.1995.tb01223.x 7652117

[B60] Ruchkin D. S. Johnson R. Grafman J. Canoune H. Ritter W. (1992). Distinctions and similarities among working memory processes: an event-related potential study. Brain Res. Cogn. Brain Res. 1, 53–66. 10.1016/0926-6410(92)90005-c 15497435

[B61] Sakai K. Rowe J. B. Passingham R. E. (2002). Active maintenance in prefrontal area 46 creates distractor-resistant memory. Nat. Neurosci. 5, 479–484. 10.1038/nn846 11953754

[B62] Santos I. M. Iglesias J. Olivares E. I. Young A. W. (2008). Differential effects of object-based attention on evoked potentials to fearful and disgusted faces. Neuropsychologia 46, 1468–1479. 10.1016/j.neuropsychologia.2007.12.024 18295286

[B63] Smith N. K. Cacioppo J. T. Larsen J. T. Chartrand T. L. (2003). May I have your attention, please: electrocortical responses to positive and negative stimuli. Neuropsychologia 41, 171–183. 10.1016/s0028-3932(02)00147-1 12459215

[B64] Stout D. M. Shackman A. J. Larson C. L. (2013). Failure to filter: anxious individuals show inefficient gating of threat from working memory. Front. Hum. Neurosci. 7:58. 10.3389/fnhum.2013.00058 23459454 PMC3586709

[B65] Valdés-Conroy B. Aguado L. Fernández-Cahill M. Romero-Ferreiro V. Diéguez-Risco T. (2014). Following the time course of face gender and expression processing: a task-dependent ERP study. Int. J. Psychophysiol. 92, 59–66. 10.1016/j.ijpsycho.2014.02.005 24594443

[B66] Vogel E. K. Machizawa M. G. (2004). Neural activity predicts individual differences in visual working memory capacity. Nature 428, 748–751. 10.1038/nature02447 15085132

[B67] Vogel E. K. McCollough A. W. Machizawa M. G. (2005). Neural measures reveal individual differences in controlling access to working memory. Nature 438, 500–503. 10.1038/nature04171 16306992

[B68] Vuilleumier P. (2005). How brains beware: neural mechanisms of emotional attention. Trends Cogn. Sci. 9, 585–594. 10.1016/j.tics.2005.10.011 16289871

[B69] Watter S. Geffen G. M. Geffen L. B. (2001). The n-back as a dual-task: P300 morphology under divided attention. Psychophysiology 38, 998–1003. 10.1111/1469-8986.3860998 12240676

[B71] Wessing I. Rehbein M. A. Postert C. Fürniss T. Junghöfer M. (2013). The neural basis of cognitive change: reappraisal of emotional faces modulates neural source activity in a frontoparietal attention network. Neuroimage 81, 15–25. 10.1016/j.neuroimage.2013.04.117 23664945

[B72] Zald D. H. (2003). The human amygdala and the emotional evaluation of sensory stimuli. Brain Res. Brain Res. Rev. 41, 88–123. 10.1016/s0165-0173(02)00248-5 12505650

[B73] Ziaei M. Peira N. Persson J. (2014). Brain systems underlying attentional control and emotional distraction during working memory encoding. Neuroimage 87, 276–286. 10.1016/j.neuroimage.2013.10.048 24185015

[B74] Zimmer H. D. (2008). Visual and spatial working memory: from boxes to networks. Neurosci. Biobehav. Rev. 32, 1373–1395. 10.1016/j.neubiorev.2008.05.016 18603299

